# High incidence of stroke in COVID-19 patients

**DOI:** 10.18632/aging.104092

**Published:** 2020-11-20

**Authors:** Jingyu Chen, Yazhou Wu, Zhi Chen, Bin Yi, Lili Zhang, Changlin Yin, Hua Feng

**Affiliations:** 1 Department of Intensive Care Unit, Taikangtongji Hospital (Wuhan), Wuhan 430040, People’s Republic of China; 2 Department of Neurosurgery, Southwest Hospital, Army Medical University (Third Military Medical University), Chongqing 400038, People’s Republic of China; 3 Department of Health Statistics, College of Preventive Medicine, Army Medical University (Third Military Medical University), Chongqing 400038, People’s Republic of China; 4 Department of Anesthesia, Southwest Hospital, Army Medical University (Third Military Medical University), Chongqing 400038, People’s Republic of China; 5 Neurology Department, Daping Hospital, Army Medical University, Chongqing 400038, People’s Republic of China; 6 Department of Intensive Care Unit, Southwest Hospital, Army Medical University (Third Military Medical University), Chongqing 400038, People’s Republic of China

**Keywords:** COVID-19, incidence rate, stroke, D-dimer

## Abstract

A retrospective analysis of 11 COVID-19 patients complicated with stroke was performed. It was found that the incidence of stroke in patients with COVID-19 was significantly higher than the average level of the general population (P=0.003), and the D-dimer levels of 11 stroke patients were significantly higher than other patients (P=0.004). The significant increase of D-dimer can be used as an early warning indicator of cerebral infarction. It is critical to have a response plan for treating acute stroke in COVID-19 patients.

## INTRODUCTION

Stroke is the second most common cause of death worldwide and the third most common cause of disability [1], while the incidence of stroke in China is the highest in the world [2]. Since the first case of coronavirus disease 2019 (COVID-19) was reported in December 2019, the long incubation period and strong infectivity have caused its rapid spread worldwide [3]. Our hospital is a specialist hospital for treating COVID-19 designated by the Chinese government. The hospital admits and treats COVID-19 patients diagnosed by various clinical methods. In this study, we reported the cases of stroke in the patients diagnosed with COVID-19 from 2020.2.13 to 2020.3.28, and we found that the incidence of stroke among these patients was significantly higher than that before the pandemic in China [4]. COVID-19 is currently a global pandemic and the occurrence of more COVID-19 patients with concurrent stroke was observed in our hospital. This report summarizes the epidemiological characteristics of these cases, and the diagnosis and treatment characteristics during the outbreak. Our report may provide valuable information, experience and lessons for treating similar patients in the future.

## RESULTS

A total of 2037 patients, between 17-99 years old, were admitted during the 45-day period from February 13, 2020 to March 27, 2020, including 918 males and 1119 females. In this study, 11 (0.54%) patients with coronavirus pneumonia were diagnosed with stroke based on neurological symptoms and confirmation by imaging. All 11 patients were local residents in Wuhan city. According to the literature [2], the average incidence rate of stroke in China is 345.1/100000/year. We found that the age-standardized incidence of stroke in patients with COVID-19 was significantly higher than the average level in China (χ^2^=8.620, P=0.003, Table 1). All 2037 patients were examined for blood D-dimer levels after admission, and the median D-dimer level in 11 patients before stroke was 889.00 ng/ml (Interquartile Range 787), which was significantly higher than that of other 2026 patients (median value 696.00 ng/ml, Interquartile Range 298.3, Z=-2.846, P =0.004, Table 2). Ten patients from this group were negative for nucleic acid tests (with an interval of at least 1 day) and positive for antibody IgG within 14 days after admission. Among them, 2 cases progressed to severe bacterial pneumonia and respiratory failure, and were given tracheal intubation ventilator to assist breathing. These two patients were not improved in consciousness and limb movement disorders, and were transferred to other hospitals for further treatment. Two other patients with no improvement in respiratory symptoms and improved neurological symptoms, as well as five patients with improved respiratory symptoms and neurological dysfunction were also transferred to other hospitals for further treatment. One patient with intracranial hemorrhage underwent bilateral ventricle drainage surgery 15 hours after hemorrhage, and continued cerebrospinal fluid drainage after surgery, who died 1 day after surgery. In addition, one patient whose nucleic acid test was still positive for the re-examination recovered from limb disability and continued to be treated in our hospital.

**Table 1:** Comparation of incidence rate.

	**This group**				**Literature [2]**					**This group**	**Literature [2]**
	**Stroke**	**Control**	**N1**	**Incidence**	**Stroke**	**Control**	**N1**	**Incidence**	**Standardized population**	**Standardized rate**	**Standardized rate**
<50 year	1	529	530	0.001887	152	288990	289142	0.00525693	**289672**	0.0018868	0.000525693
>50 year	10	1497	1507	0.006636	10	1497	1507	0.006636	**193052**	0.0066357	0.007784072
Total	**11**	**2026**	**2037**	0.0054	**1643**	**479044**	480687	0.003418025	**482724**	0.003786	0.003428479

**Table 2:** Comparation of D-dimer.

	**N**	**Mean**	**Std. Deviation**	**Minimum**	**Maximum**	**Median**	**Interquartile Range**	**Z**	**P**
stroke	11	769.64	336.04	640.0	4486.0	889.0	787.0	2.846	0.004
control	2026	694.60	4.08	219.0	1994.0	696.0	298.3		

## DISCUSSION

Increased cases of COVID-19 with concurrent stroke have been reported in this outbreak. The clinical study in New York showed that Large-Vessel stroke was presented as a feature of 5 Covid-19 patients of 33 to 49 years old. The incidence of young patients who didn't have any risk factors for strokes and mortality was much higher than expected [5]. Stroke includes cerebral hemorrhage and cerebral ischemia. About 80% of strokes are caused by ischemic cerebral infarction and 20% are caused by cerebral hemorrhage [6]. The COVID-19 patients with stroke in this study included 10 cases of cerebral ischemic stroke and 1 case of hemorrhagic stroke, which had roughly the same proportion as the general population. The age-standardized incidence of stroke in China was estimated to be 345.1/100,000 person/year [2]. The strategy to deal with the epidemic in China is comprehensive test, full collection of patients and fully covered treatment costs, thus the patients are highly cooperative and will be admitted to the hospital once they are diagnosed. There was no difference in the hospital visit rate due to factors such as age and treatment costs. The incidence rate of stroke in this study was significantly higher than the previous incidence rate in China (P=0.003), whereas existing data show that China is the Country with the highest incidence of stroke in the world [2].

Although the Severe acute respiratory syndrome coronavirus 2 (SARS-CoV-2) mainly attacks the lungs of patients, some patients with COVID-19 in China have developed symptoms of intracranial infections such as headache, epilepsy, and unconsciousness. Some even have symptoms related to COVID-19 after the first symptom of intracranial infection. Autopsy results of patients with COVID-19 showed that the brain tissue was hyperemic and edematous, and some neurons degenerated [7]. Neurologic injury has been confirmed in the infection of other CoVs such as in SARS-CoV and MERS-CoV. The researchers detected SARS-CoV nucleic acid in the cerebrospinal fluid of those patients and also in their brain tissue on autopsy [8]. Some researchers obtained the SARS-CoV-2 genome sequence in cerebrospinal fluid samples of patients with COVID-19 accompanied by neurological symptoms. In a study of 214 patients with COVID-19, neurologic symptoms were seen in 36.4% of patients and were more common in patients with severe infection (45.5%) according to their respiratory status, which included acute cerebrovascular events, impaired consciousness, and muscle injury [9]. Infection is related to the increased risk of stroke. The virus itself and the inflammatory response and release of inflammatory mediators after infection may all lead to an increased risk of stroke. Studies that used case cross-over analysis and conditional logistic regression observed that every infection type was associated with an increased likelihood of acute ischemic stroke, and respiratory infections were associated with subarachnoid hemorrhage [10]. In addition, SARS-CoV-2 was also found in autopsy brain tissue. More and more data also suggest that inflammation increases the risk of stroke. A meta-analysis included the results of 54 prospective studies confirmed that C-reactive protein concentration was associated with the long-term risk of cardiovascular events (including ischemic stroke) [11]. The C-reactive protein of all patients in this group of cases before stroke was higher than normal value. The white blood cell count is associated with ischemic events in high-risk groups, including stroke, myocardial infarction, and vascular death [12]. A prospective longitudinal cohort study also found that an increase in white blood cell count was independently associated with an increased risk of ischemic stroke among participants without a stroke history [13]. Six patients in this group had a white blood cell count higher than normal before the stroke.

Increased level of fibrinogen is significantly associated with stroke risk, increased stroke severity, and poor stroke outcomes [14]. Many studies have investigated the role of D-dimer as a marker of hypercoagulable state in stroke. In a case-control study involving 204 patients with ischemic stroke, the D-dimer levels of 104 patients with active cancer were significantly higher than that of 100 patients with inactive cancer [15], and even the D-dimer level of patients with cardiogenic stroke was also increased [16]. The D-dimer is a product of fibrinogen degradation. The elderly patients with COVID-19 accounted for the majority, especially in critically ill patients, and the D-dimer was significantly increased after the onset [17]. In this study, the D-dimer level before stroke was higher than the normal value, and also higher than the average value of that in other patients without stroke (P=0.004), indicating that patients with high D-dimer may be more likely to develop stroke. COVID-19 patients with severe infection had higher D-dimer levels than that of patients without severe infection [9]. This may be the reason why patients with severe infection are more likely to develop cerebrovascular disease. Recently, there are also some reported cases of venous sinus and deep vein thrombosis that attracted our attention. Inflammatory response caused by viral infections can lead to endothelial dysfunction and coagulation activation, which may cause thrombosis and fibrinolysis. Therefore, SARS-CoV-2 infection is associated with hypercoagulability and the potential risk of thrombosis. In the case of viral infections, antiphospholipid antibodies may be present briefly at low levels, which could be a post-infection mechanism for further extensive thrombosis. In addition to causing endothelial damage and systemic inflammatory reactions, SARS-CoV-2 infection may also promote the prethrombotic state of patients with venous occlusion, resulting in a large amount of blood clotting. However, the pathophysiology of COVID-19 complicated with thrombosis is still uncertain, and further research is needed.

In addition, it is worth mentioning that COVID-19 is highly contagious. During the outbreak, patients need to be admitted and treated to achieve the purpose of isolation. China, the United States, and many European countries with severe epidemics have adopted different strategies to admit COVID-19 patients. Since most of the medical structures for centralized treatment are temporarily established or modified, the personnel, facilities, and medicinal materials are mainly focused on the respiratory system and critical care caused by the coronavirus. As a result, there is a lack of personnel and equipment for stroke diagnosis and treatment, such as neurosurgeons, CT and nuclear magnetic resonance used for cerebral vascular imaging examination, neurosurgery operation room, and intervention room. Factors such as infection control and utilization of medical resources also need to be considered for outpatient transfer. In addition, surgery, thrombolysis, and thrombectomy for some acute strokes have strict time windows, and it is necessary to practice in advance to complete neurosurgery or endovascular treatment under enhanced protection.

In this study, the patient with cerebral hemorrhage (case 3) received surgical treatment only 15 hours after CT examination, while 2 patients with large-area infarction (case 1, 2) missed the time window for intravascular treatment. Thus, it is necessary to establish a set of plans in advance for responding to acute stroke in the hospital and to improve the related personnel and equipment configuration, or to establish a fast, safe, and controllable transfer mechanism according to different conditions in the epidemic area.

## CONCLUSIONS

The incidence of stroke in patients with COVID-19 was significantly higher than that of the general population. The significant increase of D-dimer can be used as an early warning indicator of cerebral infarction. A response plan for acute stroke is necessary.

### Limitations

This study has several limitations. First, only 11 patients were studied, which could cause biases in clinical observation. More patients, and even other reports should be considered. Second, because CT is the only feasible means for imaging examination during the outbreak of COVID-19, the condition of cerebrovascular vessels cannot be fully assessed. Third, because most patients were still hospitalized and information regarding clinical outcomes was unavailable at the time of analysis, it was difficult to assess the effect of these neurologic manifestations on their outcome, and continued observations of the natural history of disease are needed.

## MATERIALS AND METHODS

### General information

In this retrospective study, 11 patients with COVID-2019 combined with stroke who were admitted from February 13, 2020 to March 28, 2020 were included. It accounted for 0.54% of the 2037 (17-99 years old) patients with the coronavirus pneumonia admitted in the same period. The patients were 46-89 years old, with an average of 73.55 years old, including 5 males and 6 females; all patients were local residents of Wuhan and had a clear history of close contact with confirmed patients (Table 3).

**Table 3:** The general information of patients.

	**sex**	**age**	**History of hypertension**	**Smoking**	**History of diabetes**	**Stroke history**	**Long-term residence**
Case 1	female	64	No	No	No	No	Wuhan, Hubei
Case 2	female	81	No	No	No	No	Wuhan, Hubei
Case 3	female	68	Yes	No	Yes	No	Wuhan, Hubei
Case 4	male	46	Yes	Yes	No	No	Wuhan, Hubei
Case 5	female	87	No	No	No	No	Wuhan, Hubei
Case 6	male	70	No	No	No	No	Wuhan, Hubei
Case 7	male	71	No	Yes	Yes	No	Wuhan, Hubei
Case 8	female	89	Yes	No	No	No	Wuhan, Hubei
Case 9	male	84	No	No	Yes	No	Wuhan, Hubei
Case 10	male	71	Yes	No	No	Yes	Wuhan, Hubei
Case 11	female	78	Yes	No	No	No	Wuhan, Hubei

### Case inclusion criteria

1. Positive for the nucleic acid detection of the COVID-19 virus (oropharyngeal swab or blood); 2. Having been to the epidemic area or having a clear history of close contact with patients with COVID-19; 3. Confirmed diagnosis of the COVID-19 with new neurological symptoms and CT examination that showed abnormal imaging.

### Clinical manifestations

According to the report from World Health Organization(WHO) and Centers for Disease Control and Prevention (CDC), patients with COVID-19 may experience a wide range of symptoms, including fever or chills, cough, shortness of breath or difficulty to breathe, fatigue, muscle or body aches, headache, loss of taste or smell, sore throat, congestion or runny nose, nausea or vomiting, and diarrhea.

All the patients in our study had onset of cough, sputum, fever (7-21 days), and presented varying degrees of respiratory dysfunction during the course of the disease. Some critically ill patients developed respiratory failure or even multiple organ failure. In the course of treatment after diagnosis, all patients experienced neurological symptoms, five patients had sudden and persistent disturbance of consciousness, ten patients had language dysfunction and limb movement disorders. Two patients experienced a disappearance of the pupil reflection, and one patient had eye movement to the left side (Table 4).

**Table 4:** Clinical information.

	**Respiratory symptoms**	**Fever**	**Initial D-dimer (ng/ml)**	**Classification of pneumonia**	**Neurological symptoms**	**Surgery/endovascular treatment**	**Prognosis after two weeks**
Case 1	Cough, wheezing, chest tightness, difficulty breathing	No	640	Critical	Left sided hemiplegia	No	Relief of respiratory symptoms and improvement of hemiplegia, the patient was transferred to another hospital after showing negative results of two nucleic acid tests and positive result of IgG test
Case 2	Cough	No	681	Mild	Coma, right sided hemiplegia	No	Severe bacterial pneumonia, coma, no improvement in hemiplegia on the right limb. The patient was transferred to another hospital following the negative results of two nucleic acid tests and positive result of IgG test
Case 3	Cough	Yes	981	Severe	Coma	Yes	Died
Case 4	Cough, diarrhea	Yes	792	Mild	Light coma, left upper limb dyskinesia	No	Relief of respiratory symptoms, recovery of consciousness, and recovery of left upper limb muscle strength compared to admission. The patient was discharged after showing negative results of two nucleic acid tests and positive result of IgG test
Case 5	Cough, sputum, chest tightness, shortness	Yes	4486	Severe	Coma, right facial paralysis, left sided hemiplegia	No	Severe pneumonia, respiratory failure, coma, no spontaneous breathing, and no improvement, the patient was discharged after showing negative results of two nucleic acid tests and positive result of IgG test
Case 6	Cough, sputum, chest tightness	No	1481	Severe	Coma, right sided hemiplegia	No	Drowsiness and hemiplegia on the right side. lung lesions were absorbed more than before, the patient was transferred to another hospital after showing negative results of two nucleic acid tests and positive result of IgG test
Case 7	Fatigue, chest tightness	Yes	1551	Severe	Reduced right limb muscle strength	No	Limb muscle strength were recovered from the previous condition, there was still fatigue and chest tightness, the patient was transferred to another hospital after showing negative results of two nucleic acid tests and positive result of IgG test
Case 8	Expectoration, wheezing, difficulty breathing	Yes	737	Critical	Reduced left limb muscle strength	No	Muscle strength of the limb was recovered from the previous condition, there was still fatigue and chest tightness. The patient showed positive result of nucleic acid test and is under treatment
Case 9	Cough, fatigue	No	897	Common	Numbness and reduced muscle strength on right limb	No	Cough and fatigue were improved, right limb muscle strength was recovered compared to hospital admission, the patient was discharged after showing negative results of 2 nucleic acid tests and positive result of IgG test
Case 10	Cough, shortness	Yes	694	Severe	Reduced right limb muscle strength (cerebral hemorrhage surgery 11 years ago, left sided hemiplegia)	No	Cough and shortness were improved, muscle strength on the right limb was recovered compared to admission. The patient was discharged after showing negative results of 2 nucleic acid tests and positive result of IgG test
Case 11	Cough, fatigue	Yes	889	Common	numbness and dullness of left upper limb	No	Cough and fatigue were improved, the numbness and dull sensation of left upper limb were recovered compared to admission. The patient was discharged after showing negative results of 2 nucleic acid tests and positive result of IgG test

### Classification of COVID-19

Mild type: the clinical symptoms were mild and no radiological manifestations of pneumonia; common type: the clinical symptoms included fever and respiratory symptoms, and had radiological manifestations of pneumonia; severe type: patients showing respiratory distress, and pulmonary imaging showed over 50% significant progress of the lesion within 24-48 hours; critical type: patients showing respiratory failure and needed mechanical ventilation, and had shock and other organ failure.

### Diagnostic criteria for stroke

Based on the WHO criteria, stroke was defined as “rapidly developing clinical signs of focal (or global) disturbance of cerebral function, lasting more than 24 hours or leading to death, with no apparent cause other than that of vascular origin [18]. Because our medical institution was a designated hospital for treating the patients with COVID-19 during the pandemic, only plain CT scan was used for the imaging examination of the nervous system. Two neurologists and two neurosurgeons judged the stroke based on clinical manifestations and imaging studies: all patients had new neurological dysfunction after diagnosis of COVID-19, and abnormal intracranial changes were observed by CT examination, and the new neurological symptoms can be explained.

### Examination

**Figure 1:**
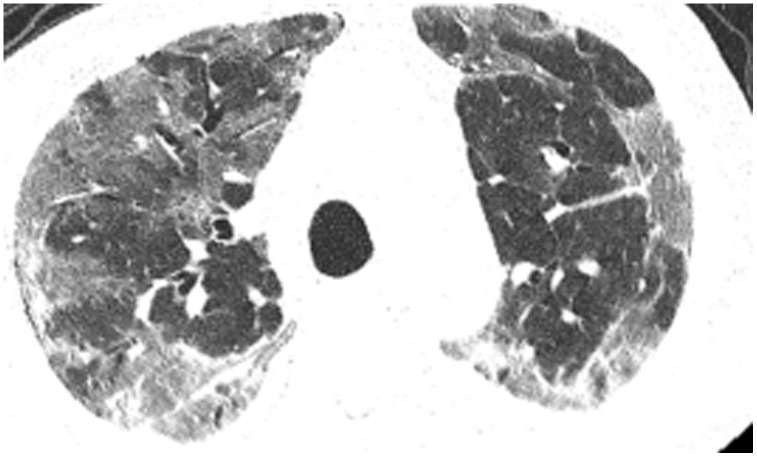
**Case 4, flaky or frosted glass like- high-density shadow in both lungs.**

**Figure 2:**
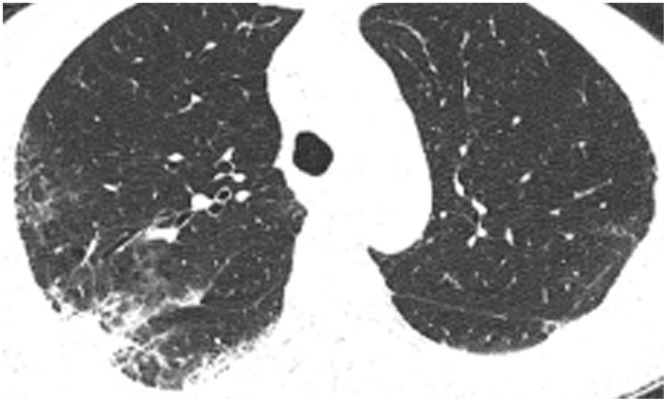
**Case 5, focal high-density shadow in right lung.**

**Figure 3:**
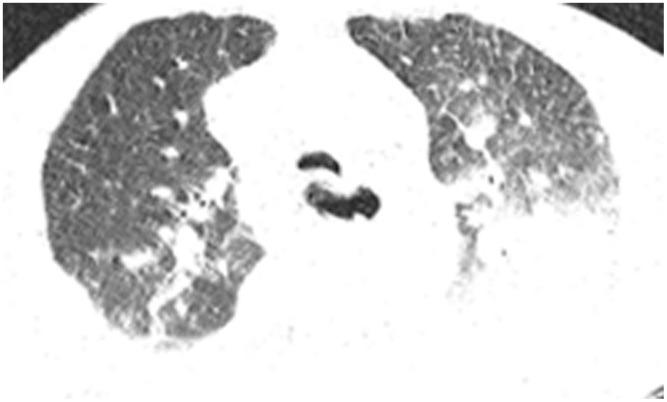
**Case 6, flaky or frosted glass like- high-density shadow in both lungs with pleural effusion in left lung.**

**Figure 4:**
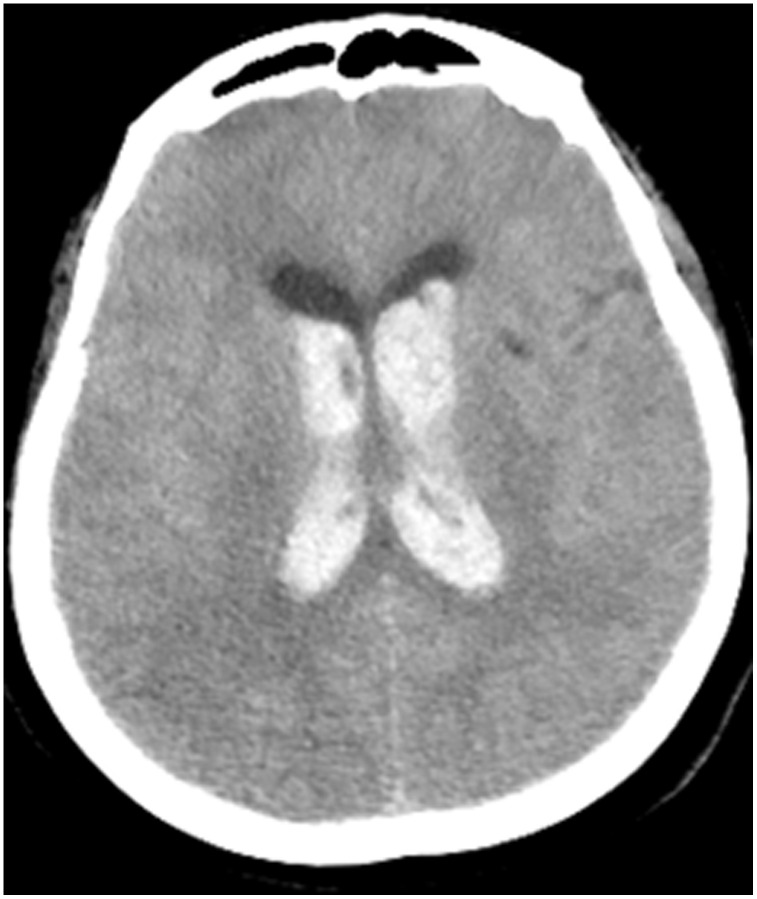
**Case 3, head CT imaging: one patient had brainstem hemorrhage with ventricular cast and hydrocephalus.**

**Figure 5:**
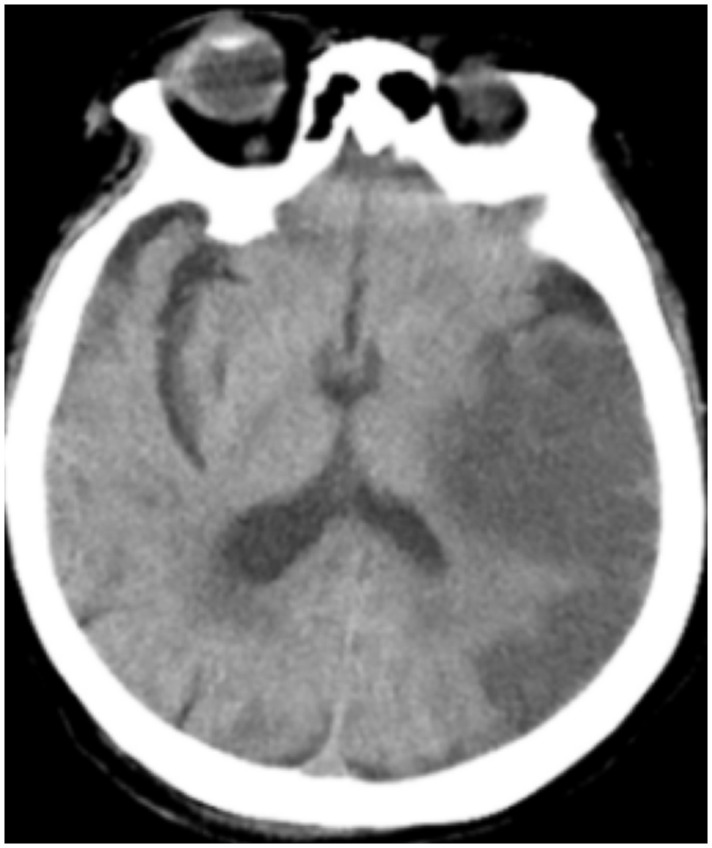
**Case 2, a large area of uniform low-density shadow on the left frontotemporal lobe.**

**Figure 6:**
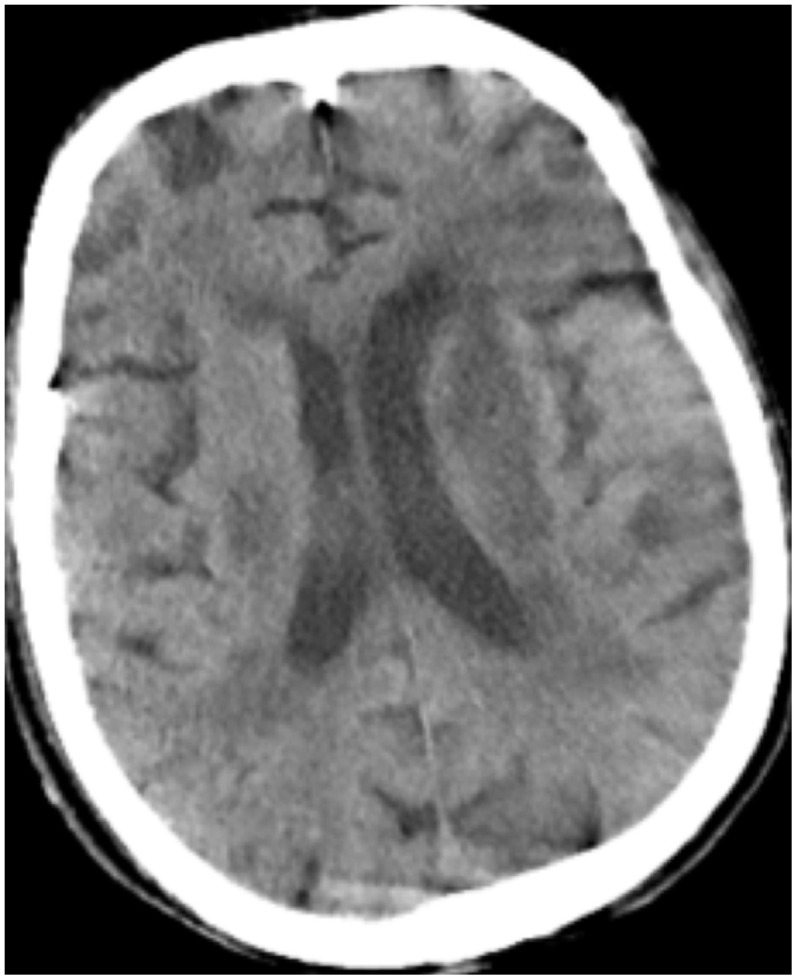
**Case 6 multiple flaky low-density shadow on the parietal lobe.**

**Figure 7:**
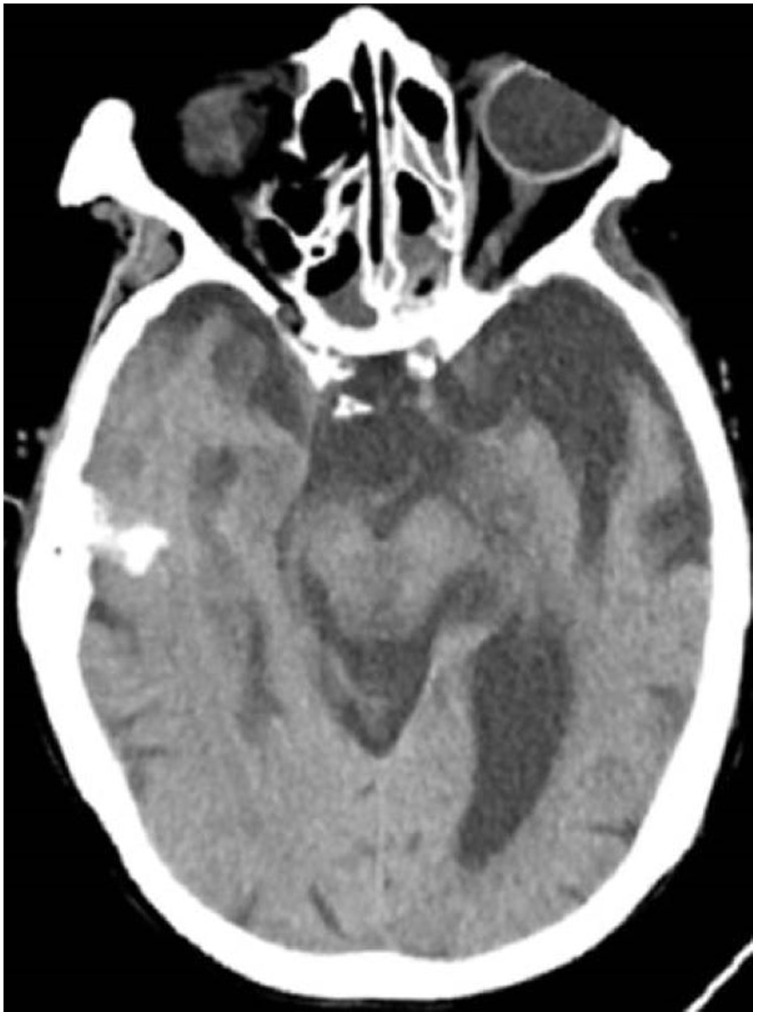
**Case 5, flaky low-density shadow in the pons.**

**Figure 8:**
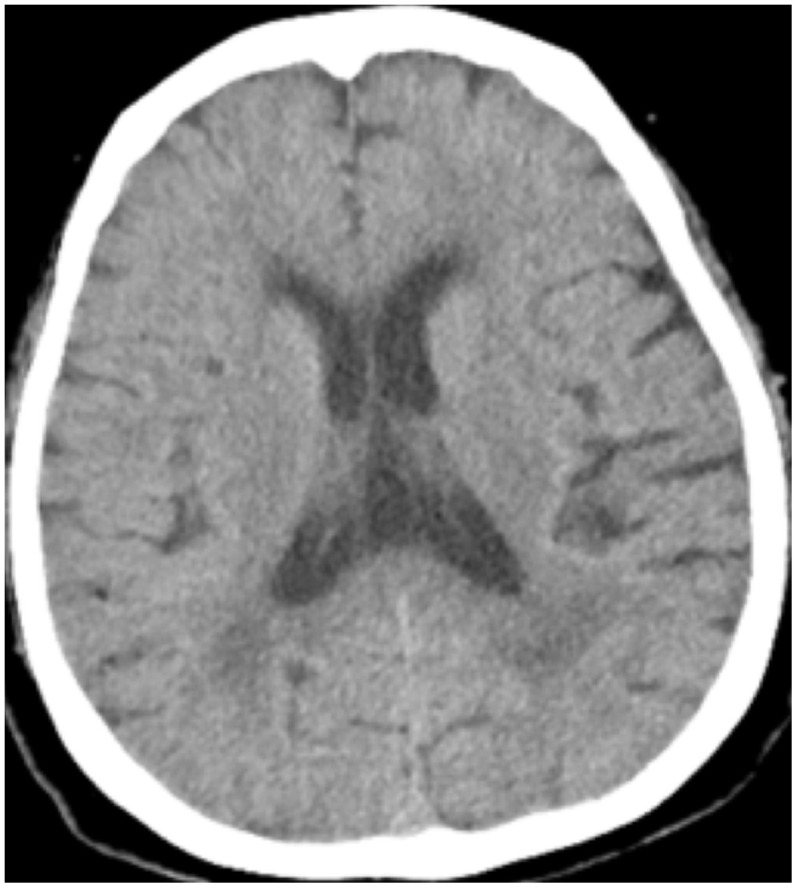
**Case 11 point-like low density shadow in basal ganglia.**

All patients were positive for the coronavirus nucleic acid test from pharyngeal swabs or blood. The blood lymphocyte counts and proportions of all patients were lower than normal (0.8×10^9^/L) at the time of admission, and the white blood cell counts and proportions of six patients were higher than normal value (10×10^9^/L). The blood D-dimer levels in all patients were higher than normal value (243 ng/mL) before neurological symptoms, and the C-reactive protein values in all patients were also higher than normal value (8000 μg/L). The chest CT of all patients showed flaky or frosted glass like- high-density shadow in lungs (Figures 1, 2), pleural effusion was seen in 2 cases (Figure 3), and no obvious abnormalities in 4 cases. The head CT imaging indicated that one patient had brainstem hemorrhage with ventricular cast and hydrocephalus (Figure 4), two patients had a large area of uniform low-density shadow on the frontotemporal lobe on the head CT (Figure 5), four patients had multiple flaky low-density shadow on the parietal lobe (Figure 6), one patient showed flaky low-density shadow in the pons (Figure 7), and three patients could see point-like low density shadow in basal ganglia (Figure 8).

All patients were given different levels of oxygen support treatment (low flow oxygen intake, high flow oxygen intake, non-invasive ventilator-assisted breathing, endotracheal intubation ventilator-assisted breathing) at different stages according to their condition after admission, and were given antiviral treatment with Abidor and Chinese medicine for symptom improvement. After the occurrence of neurological symptoms, a patient with brain stem hemorrhage with break through into the ventricle was given bilateral ventricle drainage surgery 15 hours after hemorrhage was found, and other patients with ischemic stroke were given strict blood pressure control, atorvastatin calcium, clopidogrel, and aspirin treatment, meantime the relevant indicators of embolism were closely checked.

### Statistical analysis

The age-standardized incidence of stroke was compared (Table 1). Differences between our cases and literature reports were analyzed by Pearson Chi-Square Test. For the D-dimer level of inpatients, median and Interquartile Range were used for data that were not normally distributed. Categorical variables were compared by Mann-Whitney Test. All statistical analyses were performed using SPSS 22.0.
